# Root-Associated Bacterial Community Assembly and Functional Divergence of Four Native Halophytes in the Yellow River Delta Coastal Wetland

**DOI:** 10.3390/microorganisms14092021

**Published:** 2026-09-11

**Authors:** Yunpeng Liu, Jingyi Yu, Bo Zhou, Shichang Liu, Xinping Yu, Jun Wang, Shuai Shang

**Affiliations:** 1 College of Biological and Pharmaceutical Engineering, Shandong University of Aeronautics, Binzhou 256600, China; 2 Shandong Key Laboratory of Eco-Environmental Science for the Yellow River Delta, Shandong University of Aeronautics, Binzhou 256600, China

**Keywords:** root-associated bacterial community, rhizosphere–endosphere niche, native halophytes, host filtering, Yellow River Delta coastal wetland, plant–microbe interaction

## Abstract

Coastal salt-marsh wetlands sustain diverse native halophytes whose root-associated microbiomes mediate plant adaptation to saline-alkaline environments. Host filtering and niche differentiation between rhizosphere soil and root endosphere jointly shape bacterial community assembly, yet comparative information for co-existing native halophytes in the northern Yellow River Delta remains limited. Here, we collected 80 rhizosphere soil and root tissue samples from replicated plots in the Binzhou coastal salt-marsh wetland (northern Yellow River Delta), and applied Illumina MiSeq 16S rRNA sequencing to compare bacterial communities of four native pioneer halophytes (*Phragmites australis*, *Suaeda salsa*, *Tamarix chinensis*, *Cynanchum chinense*). Rhizosphere soils had markedly higher alpha diversity than root endospheres, with *C. chinense* rhizosphere reaching the highest diversity; Proteobacteria dominated roots, whereas Actinobacteriota, Bacteroidota and Chloroflexi accumulated in rhizosphere habitats. Root bacterial communities displayed greater interspecific divergence, and rhizospheres of *S. salsa* and *P. australis* possessed enhanced nitrogen metabolism and hydrocarbon degradation. LEfSe identified host-specific biomarkers, and Chloroflexi *Subgroup_10* functioned as a core hub whereas no shared keystone genus existed across root endosphere networks, underscoring stronger host filtering in endophytic habitats. This study advances our understanding of host-driven bacterial assembly of native halophytes and provides baseline references for microbiome-assisted wetland restoration in the northern Yellow River Delta.

## 1. Introduction

The Yellow River Delta represents the youngest, most intact, and largest estuarine wetland ecosystem in China’s temperate zone, providing critical ecosystem services such as biodiversity conservation, water purification, carbon sequestration, and coastal protection [1]. However, since the early 1990s, the invasive halophyte *Spartina alterniflora* has rapidly expanded across the intertidal zone, which is closely related to its strong tolerance to flooding and salinity [2]. Its vigorous clonal propagation and high salt tolerance have enabled it to aggressively displace native vegetation, leading to a marked decline in characteristic halophytic communities, including *Phragmites australis*, *Suaeda salsa*, and *Tamarix chinensis* [3]. Consequently, these invasions have profoundly altered soil physicochemical properties and bacterial community composition [4]. In recent years, large-scale ecological restoration initiatives, driven by national and provincial policies, have been implemented in the Yellow River Delta to eradicate the invasive S. alterniflora and facilitate the recovery of native vegetation. Integrated management strategies, including mechanical mowing, hydrological regulation, and targeted habitat reconstruction, have enabled the gradual reestablishment of native plant communities, contributing to positive ecological succession at an early stage within the regional wetland ecosystem [5].

The rhizosphere bacterial community refers to the highly diverse and functionally dynamic bacterial community that colonizes the soil microenvironment immediately adjacent to plant roots. It serves as a critical biogeochemical interface mediating key plant–soil interactions, including nutrient acquisition, pathogen suppression, and stress resilience. Given its extraordinary taxonomic complexity and metabolic activity, the rhizosphere has been characterized in the scientific literature as “one of the most complex and functionally active ecosystems on Earth” [6]. The chemical environment is highly dynamic, being shaped by root exudates, soil physicochemical properties, and microbial activity [7,8]. Rhizosphere microbes are frequently described as the plant’s “second genome” owing to their profound influence on critical physiological and ecological processes, including nutrient acquisition, phytohormone modulation, abiotic stress mitigation, and pathogen suppression [3,9]. As key biotic drivers, they significantly facilitate plant establishment and shape plant community assembly in saline ecosystems. Furthermore, these microbes promote plant health and growth through multiple complementary mechanisms: (i) biosynthesis of growth-promoting phytohormones (e.g., auxins); (ii) enhancement of nutrient bioavailability and uptake, particularly of phosphorus and iron; and (iii) activation of induced systemic resistance (ISR) and/or systemic acquired resistance (SAR) [10,11]. For instance, rhizosphere microorganisms can selectively enrich beneficial bacterial communities, thereby enhancing crop performance in a dose-dependent manner, as evidenced by the observed increase in maize yield following the application of selenium nanoparticles [12,13]. Plant root exudates, including low-molecular-weight organic compounds and glucosinolate-derived metabolites, serve as key signaling molecules that modulate the assembly, structure, and functional dynamics of rhizosphere bacterial communities [14,15]. Moreover, microbial metabolites modulate plant gene expression, secondary metabolite biosynthesis, and growth-related phenotypes through feedback mechanisms [16]. Plant root-associated bacterial communities are shaped by a suite of interrelated factors, including host genotype, domestication history, ploidy level, and the presence of specific functional genes, each of which exerts a significant influence on bacterial assemblage dynamics and community assembly. Notably, certain core bacterial taxa demonstrate remarkable resilience and taxonomic stability across diverse edaphic environments [17,18].

Coastal saline-alkaline wetlands impose strong abiotic stress for plant-associated microbiomes. Pioneer native halophyte plants can reshape rhizosphere bacterial communities, which further modulates plant fitness and ecosystem self-sustainability. *P. australis* possesses well-developed aerenchyma and a strong carbon fixation ability; *S. salsa* is a typical halophytic indicator species; *T. chinensis* exhibits salt-excreting capabilities and a deep root system; and *C. chinense* demonstrates excellent ground cover and reproductive potential on mildly saline-alkaline soils. These four species represent the dominant native halophytes in northern Yellow River Delta coastal wetland. However, comparative studies on rhizosphere–endosphere bacterial communities among these co-existing native plant species remain scarce, and the inter-species differences in root-associated microecology, as well as their key driving factors, are still unclear.

In this study, we focused on the Binzhou coastal salt-marsh wetland of the northern Yellow River Delta, systematically comparing the bacterial community structures, diversity, and composition in rhizosphere soil and root endosphere of four typical native halophytes, including the *P. australis*, *S. salsa*, *T. chinensis*, and *C. chinense*. The aim was to clarify the assembly patterns of root-associated microbes and plant-driven filtering effects among co-existing native halophytes. Our findings provide theoretical support and potential bacterial resources for plant–microbe synergistic restoration strategies in degraded saline-alkaline wetland ecosystems.

Based on the above research gaps, we put forward two core hypotheses of this study:

(i) The rhizosphere soil and root endosphere constitute distinct micro-habitat niches, driving significant differentiation in taxonomic composition, alpha-diversity and functional profiles of bacterial communities for coastal halophytes;

(ii) The four native pioneer halophytes recruit host-specific bacterial assemblages with distinct biomarker taxa and functional potential. We systematically analyzed bacterial community diversity, taxonomic composition, host-specific biomarkers, co-occurrence networks, and functional prediction across root and rhizosphere samples of the four halophytes to verify the above hypotheses.

## 2. Materials and Methods

### 2.1. Study Area

The study site is located in the coastal salt-marsh wetland near Binzhou Port (Taoer River estuary, northern Yellow River Delta), Binzhou, Shandong Province, China. This area belongs to the northern margin of the modern Yellow River Delta, with a warm-temperate continental monsoon climate. Mean annual temperature is approximately 12.1 °C; annual precipitation is 530–630 mm, and the evaporation–precipitation ratio is around 3.5. The soil type is coastal saline soil. Historically, this tidal flat experienced *S. alterniflora* invasion, and large-scale invasive-plant removal measures (mechanical mowing combined with hydrological regulation) were implemented for ecological restoration. After eradication, native halophyte communities have naturally regenerated on the site. Sampling was performed in June 2026 during the peak growing season of local halophytes. Four dominant native halophyte species were selected within continuous restored vegetation zones, paired rhizosphere-soil and root-tissue samples were collected for each species.

CLG: *T. chinensis* root tissue; CLS: *T. chinensis* rhizosphere soil

LWG: *P. australis* root tissue; LWS: *P. australis* rhizosphere soil

JPG: *S. salsa* root tissue; JPS: *S. salsa* rhizosphere soil

ERTG: *C. chinense* root tissue; ERTS: *C. chinense* rhizosphere soil.

For each community type, ten replicate plots (20 m × 20 m) were installed, with inter-plot distances exceeding 30 m to minimize edge effects and spatial autocorrelation.

### 2.2. Rhizosphere Soil Sampling

Three to five healthy individual plants were randomly selected from each experimental plot, and rhizosphere soil and root tissues were synchronously collected from the same plant to form paired samples. Rhizosphere soil was first collected using the root-shaking method: after removing loosely attached and macro-aggregated soil particles, the soil tightly adhering to the root surface (within approximately 2 mm of the root epidermis) was harvested. The attached soil was gently brushed off with a sterile brush, pooled and homogenized to generate a composite rhizosphere sample per plot.

After rhizosphere soil collection, the intact fine root systems of the same plants were intercepted, repeatedly rinsed with sterile phosphate-buffered saline (PBS) to remove residual surface soil particles, and cut into small root fragments. All root fragments from one plot were mixed as a composite root sample.

Both rhizosphere soil and root tissue composite samples were immediately transferred into sterile centrifuge tubes, flash-frozen in liquid nitrogen, and stored at −80 °C prior to DNA extraction. In total, 40 rhizosphere soil samples and their 40 matched root samples were obtained for subsequent sequencing analysis.

### 2.3. DNA Extraction and High-Throughput Sequencing

Total genomic DNA was extracted separately from rhizosphere soil and root tissue samples using the PowerSoil^®^ Pro DNA Isolation Kit (QIAGEN, Hilden, Germany) following the manufacturer’s standardized protocols. For rhizosphere soil samples, 0.5 g fresh soil was directly subjected to lysis and DNA purification operations. For root tissue samples, fresh root fragments were fully ground into powder with liquid nitrogen prior to lysis to improve DNA extraction efficiency and yield.

DNA concentration and purity were spectrophotometrically determined using a NanoDrop One microvolume UV-Vis spectrophotometer (Thermo Fisher Scientific, Waltham, MA, USA), and DNA integrity was verified by electrophoresis on a 1% (*w*/*v*) agarose gel stained with GelRed. The hypervariable V3-V4 region of the bacterial 16S rRNA gene was amplified via polymerase chain reaction (PCR) using the forward primer 338F (5′-ACTCCTACGGGAGGCAGCA-3′) and reverse primer 806R (5′-GGACTACHVGGGTWTCTAAT-3′). PCR thermal cycling conditions were set as follows: initial denaturation at 95 °C for 3 min; 25 cycles of denaturation at 95 °C for 30 s, annealing at 55 °C for 30 s, extension at 72 °C for 30 s; with a final extension step at 72 °C for 5 min.

After PCR amplification, amplicons were purified using AMPure XP magnetic beads (Beckman Coulter, Brea, CA, USA) to remove primer dimers and non-specific fragments. The purified products underwent terminal repair, adapter ligation and fragment screening to construct Illumina (San Diego, CA, USA) sequencing libraries. All qualified libraries were fluorometrically quantified using the Qubit™ dsDNA HS Assay Kit (Thermo Fisher Scientific, Waltham, MA, USA), normalized to equal molar concentrations and pooled together. Paired-end sequencing (2 × 300 bp) was performed on the Illumina MiSeq platform at Beijing Biomarker Technologies Co., Ltd. (Beijing, China). Raw sequencing data were converted into FASTQ format files containing read sequences and corresponding quality scores for subsequent bioinformatic analysis.

### 2.4. Processing of Sequence Data and Bioinformatics Analysis

Sequencing data were processed using QIIME2 (version 2020.6). Quality control, denoising, paired-end read merging, and amplicon sequence variant (ASV) inference were performed with the DADA2 plugin with fixed parameters as follows: Reads with an average Phred quality score <20 were filtered out; primer sequences were trimmed, and reads containing ambiguous N bases were discarded during quality control. DADA2 error correction was executed with the built-in error training model; paired reads were merged with a minimum overlapping length of 12 bp, and overlaps carrying mismatches were discarded. The target V3–V4 amplicon of the 16S rRNA gene is approximately 460 bp long; only merged sequences ranging from 420 bp to 490 bp were reserved for taxonomic assignment. To eliminate sequencing depth bias for alpha-diversity comparison, all samples were uniformly rarefied to 38,110 clean reads per sample, which was the minimum valid read number among all 80 samples. Bacterial ASVs were taxonomically classified against the SILVA database (v138). Sequences identified as mitochondrial, chloroplast, or non-target were removed prior to downstream analysis. Alpha-diversity indices, including Shannon diversity, Chao1 richness, and Pielou’s evenness, were computed from the rarefied ASV abundance table using the vegan package (v2.6-4) in R (v4.2.3). Beta-diversity was assessed via principal coordinates analysis (PCoA) based on Bray–Curtis dissimilarity, and the statistical significance of community composition differences among groups was evaluated using permutational multivariate analysis of variance (PERMANOVA, 999 permutations). Biomarker taxa distinguishing plant community groups were identified using linear discriminant analysis effect size (LEfSe, v1.1.2), with an LDA score threshold >3.5 and a significance level of *p* < 0.05 (Kruskal–Wallis test followed by pairwise Wilcoxon rank-sum tests with Benjamini–Hochberg correction). Functional potential of bacterial communities was predicted using FAPROTAX (v1.4.7). Associations between rhizosphere microbial community structure (constrained ordination scores) and soil physicochemical variables were examined via redundancy analysis (RDA) or canonical correspondence analysis (CCA), depending on gradient length; the statistical significance and contribution of individual environmental variables were assessed using the envfit function (999 permutations). All continuous variables are reported as mean ± standard deviation. Differences in soil physicochemical properties and alpha-diversity indices across plant communities were analyzed using one-way analysis of variance (ANOVA) followed by Tukey’s honestly significant difference (HSD) post hoc test (SPSS v26.0); statistical significance was defined as α = 0.05. Prior to one-way ANOVA, the Shapiro–Wilk test was used to assess the normality of each group’s diversity data, and Levene’s test was conducted to verify homogeneity of variances. For datasets that violated the normality or homogeneity assumptions, Wilcoxon rank-sum test with Bonferroni correction was adopted for pairwise comparisons instead. All figures were generated using the ggplot2 package (v3.4.1) in R. Bacterial co-occurrence networks were constructed at the genus taxonomic level. First, genera with total relative abundance lower than 0.01% across all samples were removed to eliminate interference from rare taxa. Spearman’s rank-correlation coefficients were calculated for pairwise genus abundance relationships, and multiple testing correction was conducted via the FDR method. Only correlation pairs with |r| > 0.7 and corrected *p* < 0.05 were retained to build the network. The network was visualized using Gephi (v0.9.2) software. Key topological indicators including node degree, betweenness centrality, closeness centrality and modularity were automatically calculated. We further divided the dataset into root compartment subgroups and rhizosphere soil subgroups, as well as four plant species subgroups, to compare variations in average degree, modularity and hub node characteristics among different groups.

## 3. Results

### 3.1. Analysis of ASV Abundance in Plant Roots and Rhizosphere Soil Microbiota

**Figure 1:**
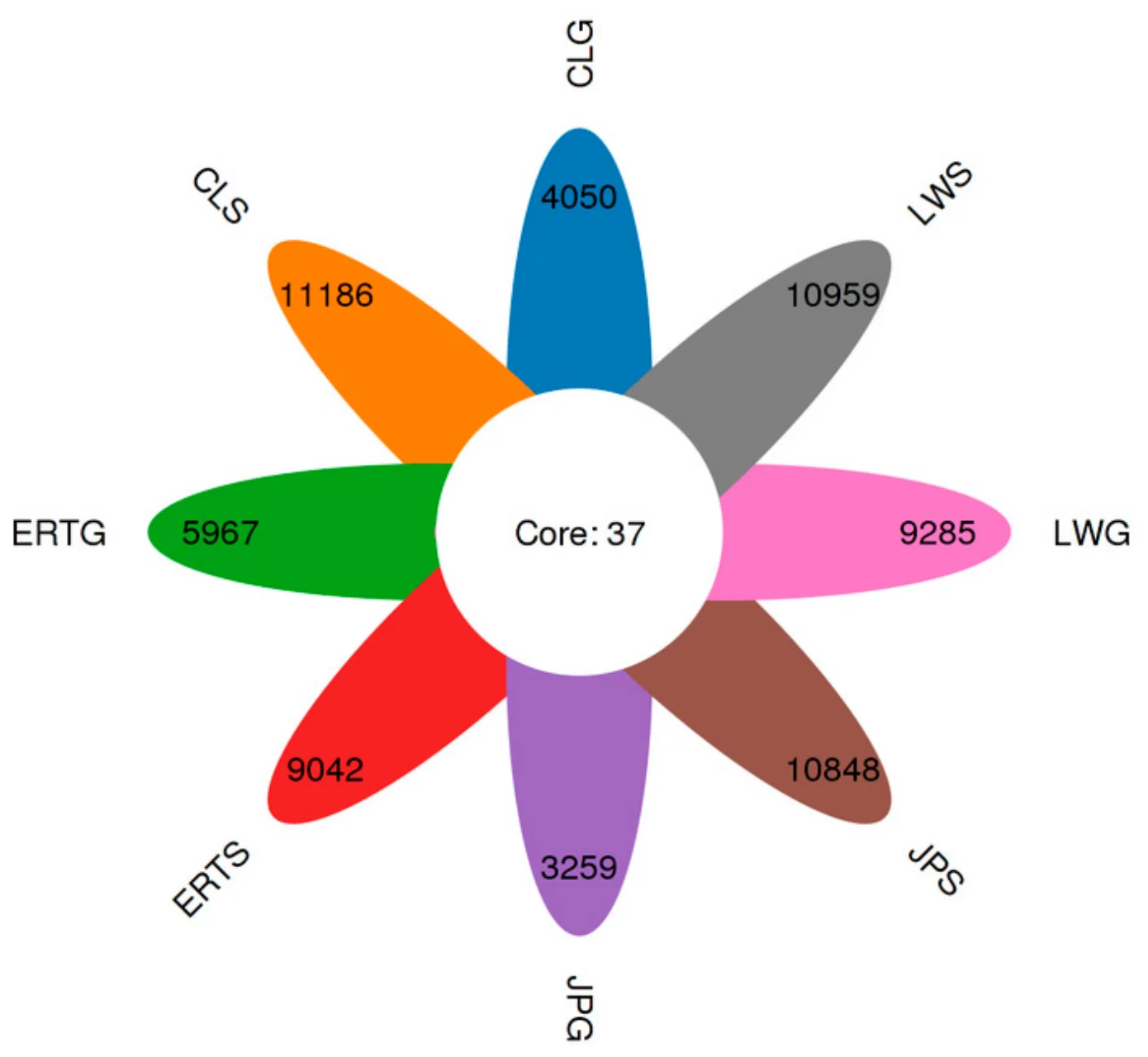
Venn diagram illustrating the distribution of amplicon sequence variants (ASVs) across rhizosphere soil and root samples of four plant species. Numerical values denote the number of ASVs uniquely present in each compartment or shared among two or more compartments. Abbreviations: CLS = *T. chinensis* rhizosphere soil, CLG = *T. chinensis* root; LWS = *P. australis* rhizosphere soil, LWG = *P. australis* root; JPS = *S. salsa* rhizosphere soil, JPG = *S. salsa* root; ERTS = *C. chinense* rhizosphere soil, ERTG = *C. chinense* root.

This study generated a total of 5,026,688 raw read pairs. After quality control and paired-end read assembly (also referred to as merging), 4,462,816 clean reads were obtained. The minimum number of clean reads per sample was 38,110, and the average was 55,785. Based on these high-quality sequences, ASV clustering was carried out. Heatmap analysis (Figure 1) showed significant differences in ASV composition among different media types. A total of 11,186 unique ASVs were identified in CLS, 4050 in CLG, 10,959 in LWS, 9285 in LWG, 10,848 in JPS, 3259 in JPG, 9042 in ERTS, and 5967 in ERTG. Notably, only 37 ASVs were shared across all eight samples, which indicates that bacterial communities in different plants, rhizosphere soils, and root microhabitats are highly distinctive, with very few species capable of widespread distribution across all tested media.

### 3.2. Analysis of Plant Rhizosphere Soil and Root Samples Bacterial Community Structure

**Figure 2:**
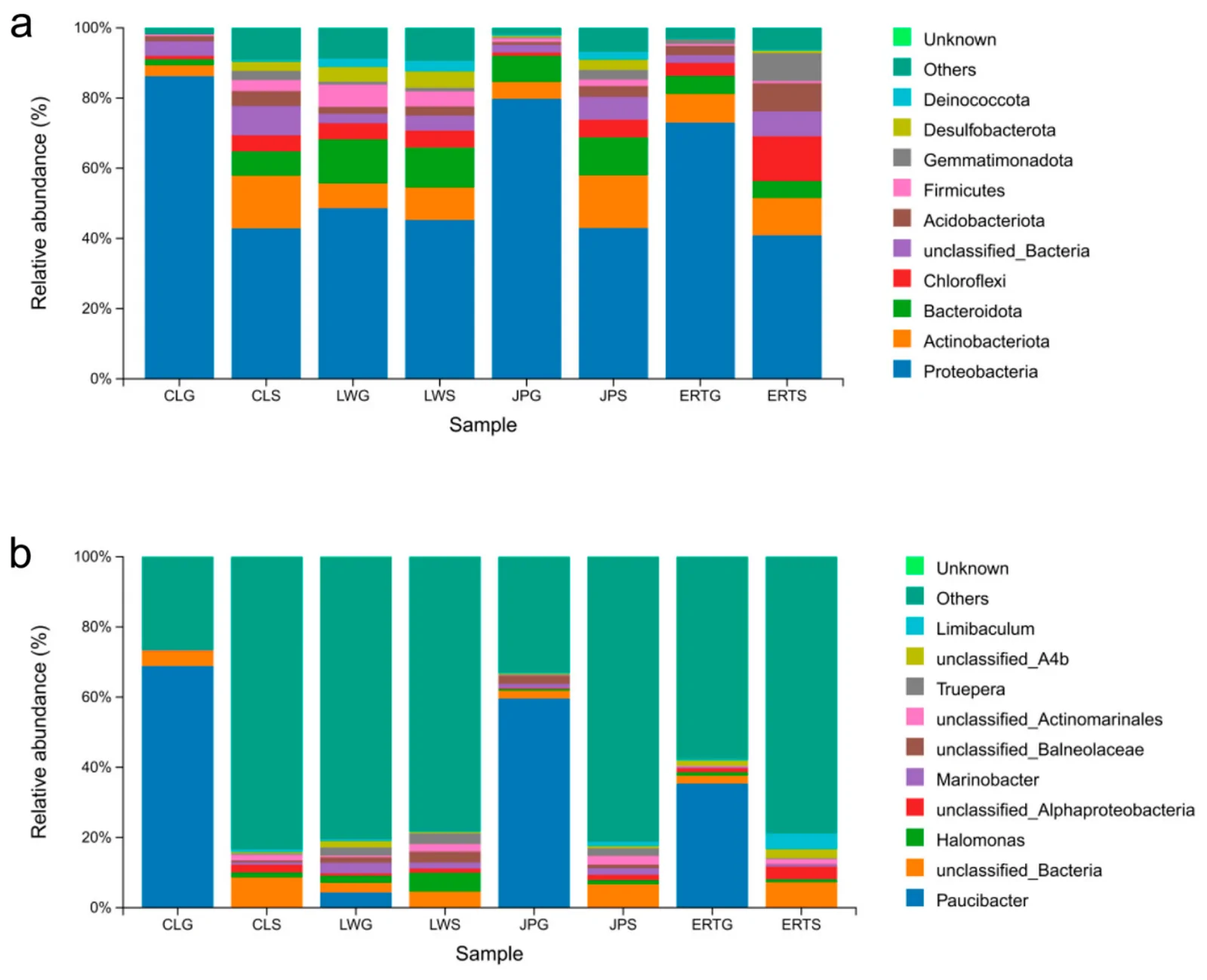
Bar charts depicting the relative abundance (%) of bacterial communities in rhizosphere soil and root samples across four plant species, at both phylum and genus taxonomic levels. (**a**) Phylum-level composition; (**b**) genus-level composition. Bacterial phyla are indicated in the legend; the *y*-axis displays relative abundance as a percentage. Abbreviations: CLS = *T. chinensis* rhizosphere soil, CLG = *T. chinensis* root; LWS = *P. australis* rhizosphere soil, LWG = *P. australis* root; JPS = *S. salsa* rhizosphere soil, JPG = *S. salsa* root; ERTS = *C. chinense* rhizosphere soil, ERTG = *C. chinense* root.

At the phylum level (Figure 2a), Proteobacteria emerged as the dominant phylum in all samples, with relative abundances consistently exceeding 40% across all eight groups. Notably, both CLG and JPG displayed the highest abundances of Proteobacteria, each surpassing 80%. Actinobacteriota, Bacteroidota, and Chloroflexi were the subsequent most abundant phyla and were extensively distributed across all samples. Firmicutes, Acidobacteriota, Desulfobacterota, Gemmatimonadota, and Deinococcota also formed major components of the bacterial communities. Remarkable disparities were detected in the bacterial community structures between root systems and rhizosphere soils: Proteobacteria generally occupied a larger proportion in plant roots compared to their corresponding rhizosphere soils. In contrast, Actinobacteriota, Bacteroidota, and several other phyla demonstrated higher abundances in rhizosphere soils. Gemmatimonadota exhibited relatively high abundance only in ERTS, underscoring the selective filtering effect of *Cynanchum chinense* rhizosphere soil on specific bacterial taxa.

At the genus level (Figure 2b), the low-abundance merged group “Others” held an absolute majority dominance in all samples, with relative abundances surpassing 70% in most instances. *Paucibacter* ranked second, exhibiting high abundance solely in CLG, JPG, and ERTG, and being almost undetectable in all rhizosphere soil samples. *Halomonas*, *Limibaculum*, *Truepera*, and *Marinobacter*, which are salt-tolerant genera, were detected in all saline-alkaline soil samples. Unclassified_Bacteria, unclassified_Actinomarinales, and unclassified_Balneolaceae showed higher relative abundance across all rhizosphere soil compartments. The dominant genera varied significantly across different microhabitats: *Paucibacter* was significantly enriched in root endospheres, whereas rhizosphere soils contained various unclassified bacteria and multiple rare salt-tolerant genera. The bacterial compositions of roots and rhizosphere soils differed significantly among the four plant species, with distinct dominant genera with unique abundance patterns in the roots of each plant species.

### 3.3. Alpha Diversity Analysis of Plant Rhizosphere Soil and Root Samples Bacterial Communities

**Figure 3:**
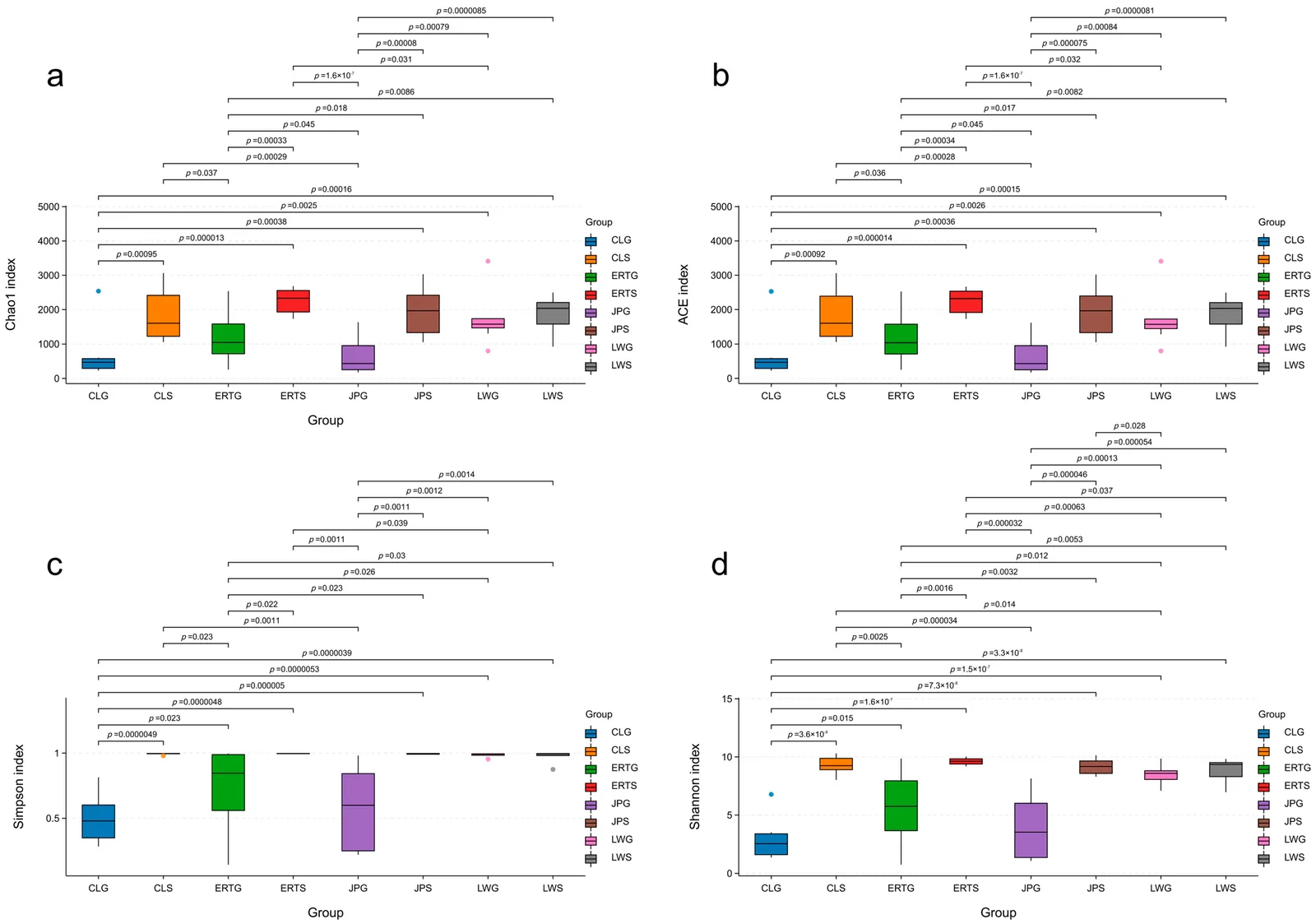
Alpha diversity of bacterial communities in rhizosphere soil and root samples across the four plant species. (**a**) Chao1 index; (**b**) ACE index; (**c**) Simpson index; (**d**) Shannon index. Box plots depict the distribution of diversity indices across sample groups. Statistical significance among groups was assessed using pairwise Wilcoxon rank-sum tests with Bonferroni correction; significant differences (*p* < 0.05) are indicated above corresponding box plot pairs. Abbreviations: CLS = *T. chinensis* rhizosphere soil, CLG = *T. chinensis* root; LWS = *P. australis* rhizosphere soil, LWG = *P. australis* root; JPS = *S. salsa* rhizosphere soil, JPG = *S. salsa* root; ERTS = *C. chinense* rhizosphere soil, ERTG = *C. chinense* root.

To elucidate the differences in bacterial community alpha diversity between the roots and rhizosphere soils of the four plant species in the Yellow River Delta, Chao1, ACE, Simpson, and Shannon indices were calculated and subjected to inter-group statistical comparisons. The Chao1 (Figure 3a) and ACE (Figure 3b) indices revealed highly congruent trends, with median values consistently higher in all rhizosphere soil samples (CLS, ERTS, JPS, LWS) than in their corresponding root samples (CLG, ERTG, JPG, LWG), indicating markedly greater species richness in the rhizosphere. Among all groups, ERTS exhibited the highest species richness, followed by JPS, whereas JPG and CLG recorded the lowest values. Pairwise comparisons were predominantly significant at *p* < 0.05, with numerous pairs reaching highly significant levels, confirming that bacterial richness in rhizosphere soils significantly surpasses that in roots. In addition, species richness varied notably among plant species, with rhizospheres of *C. chinense* and *S. salsa* harboring greater bacterial richness than those of tamarisk and reed. The Simpson index results (Figure 3c) showed comparable median values across the four rhizosphere soil types, whereas greater differentiation was observed among the root types, with LWG displaying the highest median and CLG the lowest. For each plant species, the median Simpson values of rhizosphere soils consistently exceeded those of roots. Most inter-group comparisons were significant at *p* < 0.05, with several pairs exhibiting highly significant differences, thereby demonstrating statistically distinct bacterial community evenness between roots and rhizosphere soils. The Shannon index (Figure 3d) exhibited a trend consistent with the Chao1 and ACE indices, as median values for all rhizosphere soils surpassed those of their paired root samples, suggesting greater overall bacterial community diversity in the rhizosphere. The majority of inter-group comparisons yielded *p* < 0.05, indicating significant diversity differences among groups. Collectively, these results demonstrate a clear differentiation in alpha diversity between roots and rhizosphere soils, with the latter consistently showing elevated species richness, community evenness, and overall diversity compared to the corresponding plant roots. Furthermore, the alpha diversity levels varied considerably among the four plant species investigated. These consistent differences in alpha diversity between rhizosphere soil and root endosphere preliminarily support our first hypothesis regarding niche-driven bacterial community differentiation.

**Figure 4:**
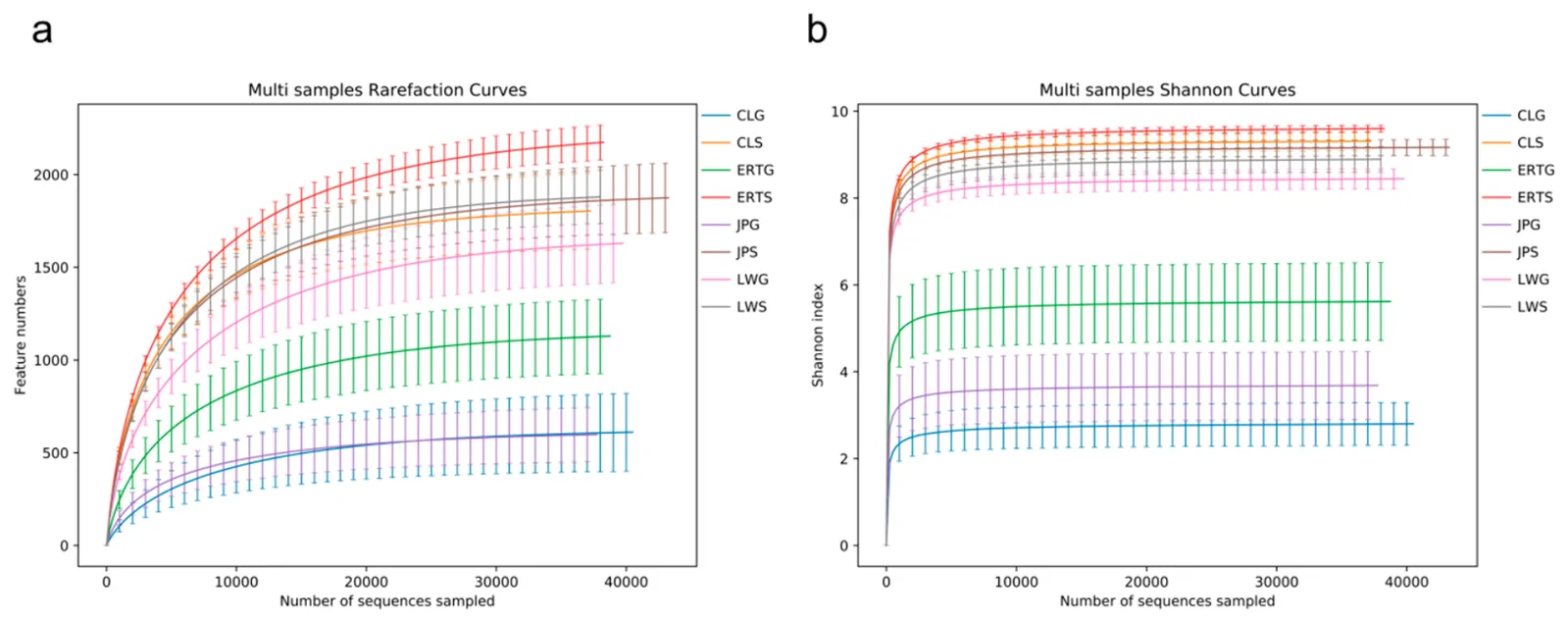
Rarefaction curves illustrating bacterial alpha diversity in rhizosphere- and root-associated soils across four plant species. Panel (**a**) shows the observed species (OTU) rarefaction curve; panel (**b**) presents the Shannon index rarefaction curve. The *x*-axis denotes sequencing depth (number of reads per sample); a plateau in the curve indicates that sequencing effort is sufficient to capture the majority of extant taxa, whereas the asymptotic height of the curve reflects species richness and overall community diversity. Abbreviations: CLS = *T. chinensis* rhizosphere soil, CLG = *T. chinensis* root; LWS = *P. australis* rhizosphere soil, LWG = *P. australis* root; JPS = *S. salsa* rhizosphere soil, JPG = *S. salsa* root; ERTS = *C. chinense* rhizosphere soil, ERTG = *C. chinense* root.

Dilution curves (Figure 4) effectively illustrate both the adequacy of sequencing depth and the dynamics of bacterial community composition across increasing sequencing effort. As sequencing depth increases, all curves exhibit an initial steep rise, followed by a marked deceleration and eventual asymptotic plateau, indicating that current sequencing depth has captured the majority of detectable bacterial taxa within each sample group. This saturation behavior confirms sufficient sequencing coverage, thereby ensuring robust and representative quantification of alpha diversity across all groups and supporting rigorous downstream statistical analyses. Moreover, the relative vertical positioning of the curves aligns closely with corresponding box plots of alpha diversity indices, reinforcing consistency in diversity estimation. For each plant species, the dilution curves for rhizosphere soils (CLS, ERTS, JPS, LWS) consistently exceed those for matched root tissues (CLG, ERTG, JPG, LWG), reflecting higher taxon detection rates and greater bacterial richness and evenness in rhizosphere environments relative to endophytic compartments. Among all samples, ERTS displays the highest curve, signifying maximal observed species richness and diversity; conversely, CLG exhibits the lowest curve, indicating the most depauperate bacterial community. Cross-species comparison reveals that rhizosphere soils of *C. chinense* and *S. salsa* yield higher dilution curves than those of *T. chinensis* and *P. australis*. In contrast, root-associated samples uniformly display lower curve elevations, with CLG (*T. chinensis* roots) and JPG (*S. salsa* roots) showing the lowest levels of taxon detection. Error bars reflect modest intra-group variability and technical replication fluctuations; however, the hierarchical stratification among sample types and plant species remains statistically consistent and biologically interpretable.

### 3.4. Beta Diversity Analysis of Plant Rhizosphere Soil and Root Samples Bacterial Communities

**Figure 5:**
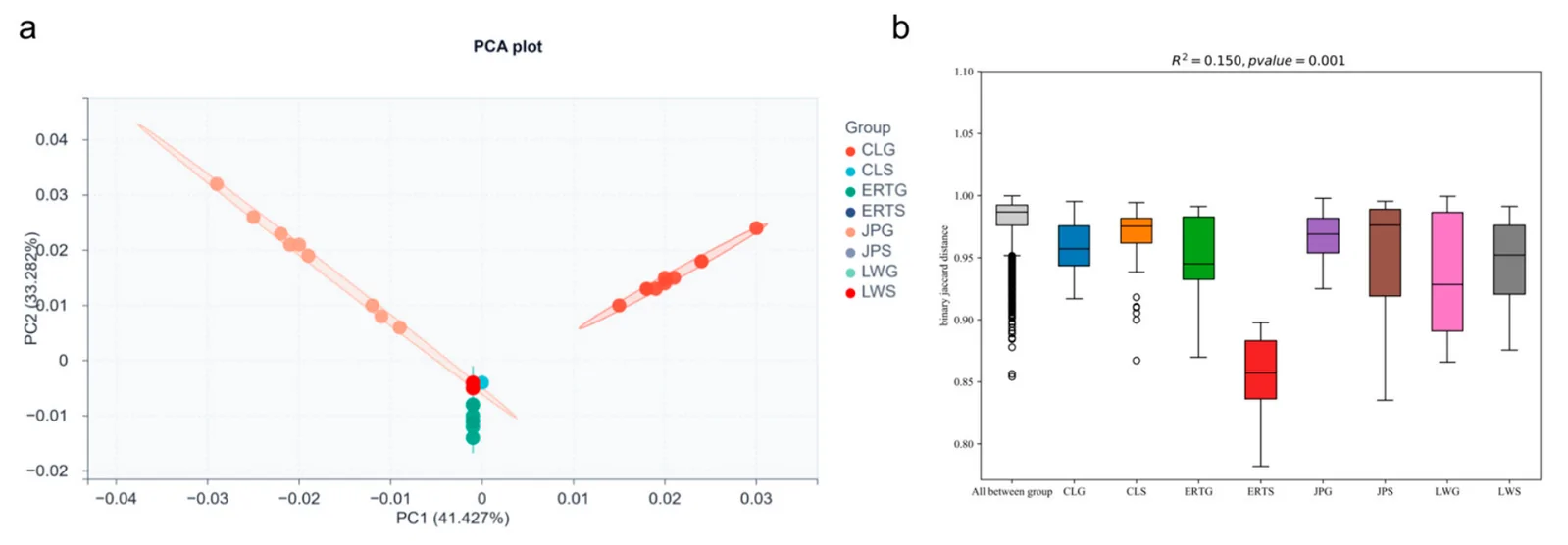
β-diversity analysis of bacterial communities based on Jaccard distance. (**a**): PCA plot, where PC1 and PC2 together explain most of the total variation in bacterial communities; different colored dots represent samples from distinct groups, with closer spatial proximity along the axes indicating higher similarity in community composition. (**b**): Box plots of inter-group and intra-group distances based on Jaccard distance; the box represents the median and quartiles of the distance distribution, whiskers indicate minimum and maximum values, and dots represent outliers. Abbreviations: CLS = *T. chinensis* rhizosphere soil, CLG = *T. chinensis* root; LWS = *P. australis* rhizosphere soil, LWG = *P. australis* root; JPS = *S. salsa* rhizosphere soil, JPG = *S. salsa* root; ERTS = *C. chinense* rhizosphere soil, ERTG = *C. chinense* root.

Principal component analysis (PCA) was employed to assess beta-diversity patterns in bacterial community composition across sample types (Figure 5a). Axes PC1 and PC2 collectively accounted for 74.709% of the total variation (PC1: 41.427%; PC2: 33.282%), indicating that the ordination effectively captured major compositional gradients among samples. Permutational multivariate analysis of variance (PERMANOVA), conducted using the binary Jaccard distance matrix (Figure 5b), revealed statistically significant differences in bacterial community structure across sample categories (R^2^ = 0.150, *p* = 0.001), confirming distinct community assemblages associated with root tissue, rhizosphere soil, and plant species. In the PCA ordination, root samples exhibited clear separation along PC1, with JPG roots clustering in the negative PC1 quadrant, ERTG and CLG roots proximal to the origin, and LWG roots occupying the positive PC1 quadrant—demonstrating consistent differentiation among root-associated bacterial communities across plant taxa. In contrast, rhizosphere soil samples (CLS, ERTS, JPS, and LWS) displayed substantial overlap and were predominantly centered near the origin, reflecting higher compositional similarity across rhizosphere habitats. Notably, for each plant species, root and corresponding rhizosphere soil samples occupied non-overlapping regions in ordination space, underscoring systematic divergence in bacterial community structure between these two habitat compartments. PERMANOVA-derived within-group median pairwise distances further supported this pattern: the ERTS group exhibited the lowest median intra-group distance (indicating highest internal homogeneity), whereas all other rhizosphere soil and root sample groups showed comparatively higher median distances, consistent with greater community dispersion. Crucially, the overall median inter-group distance significantly exceeded the median intra-group distances for most groups (*p* < 0.05, pairwise PERMDISP), confirming that compositional differences among sample categories are substantially larger than variation within categories—a finding indicative of robust, biologically meaningful partitioning of bacterial communities across plant-associated niches. The clear separation of root and rhizosphere samples in PCA ordination demonstrates distinct bacterial community compositions between these two microhabitats. To further quantify the relative contributions of compartment (rhizosphere vs. root) and plant species to community variation, stratified PERMANOVA was performed. The compartment factor explained a higher proportion of total bacterial community variation than the plant species factor, providing statistical evidence for stronger community differentiation between rhizosphere and root endosphere habitats than among different host plants. This result validates the proposed pattern of rhizosphere–endosphere niche segregation.

### 3.5. Significance Analysis of Intergroup Differences

**Figure 6:**
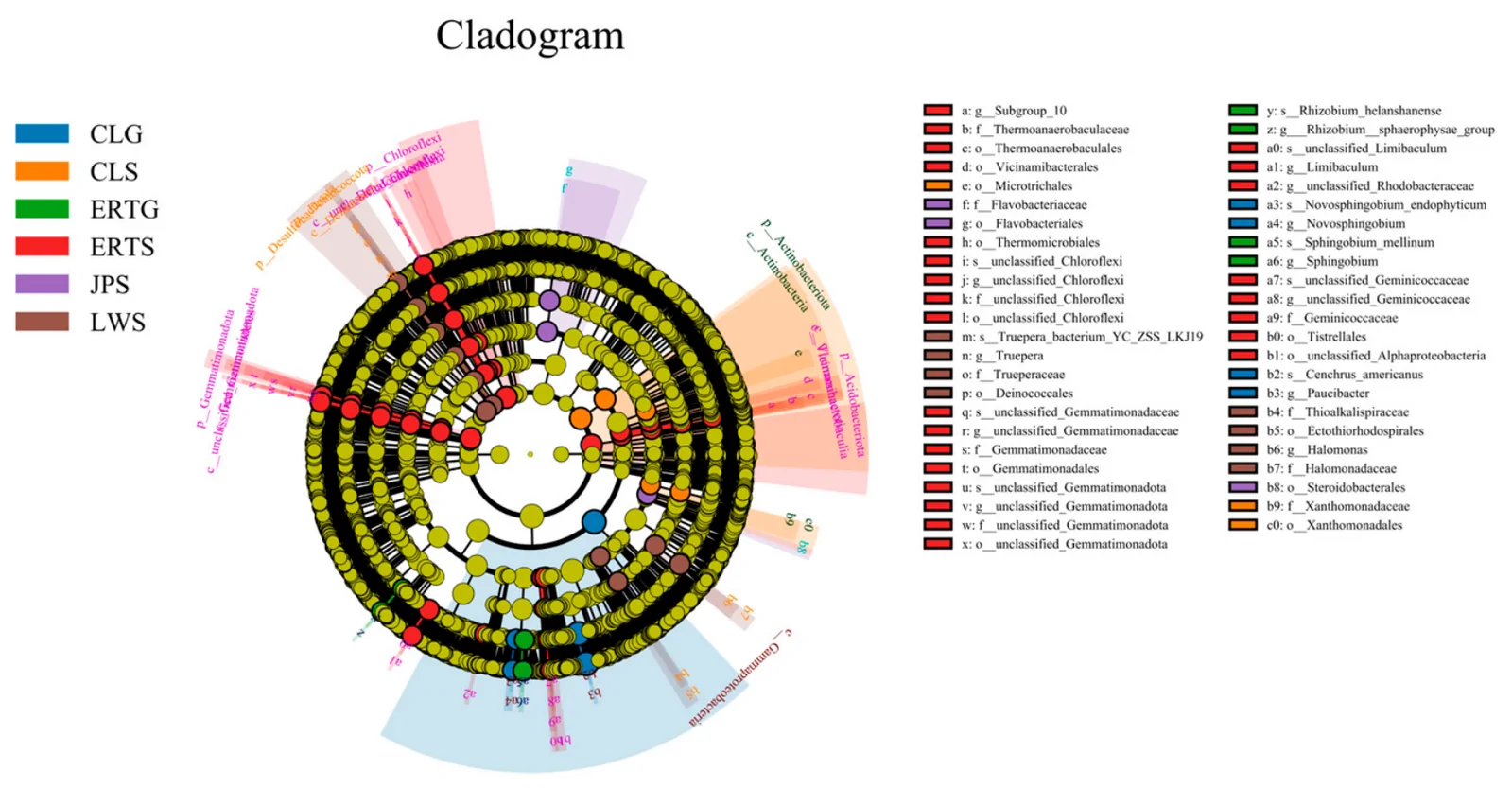
Evolutionary branch diagram of differentially abundant bacterial taxa in four plant rhizosphere soil and root samples based on LEfSe analysis. The analysis was set with LDA ≥ 4, and only significantly enriched bacterial taxa reaching this threshold were assigned group-specific colors. No significant differential taxa met the threshold for *S. salsa* roots (JPG) and *P. australis* roots (LWG), so no corresponding colored branches are shown; these two groups are not included in the legend. The phylogenetic tree is organized from inner to outer circles representing phylum, class, order, family, genus, and species levels, respectively. The color blocks in the outer ring indicate the group in which each taxon is enriched. Abbreviations: CLS = *T. chinensis* rhizosphere soil, CLG = *T. chinensis* root; LWS = *P. australis* rhizosphere soil, LWG = *P. australis* root; JPS = *S. salsa* rhizosphere soil, JPG = *S. salsa* root; ERTS = *C. chinense* rhizosphere soil, ERTG = *C. chinense* root.

The cladogram revealed that the ERTS group contributed the highest number of significantly enriched taxa (Figure 6). Detailed information for all LEfSe-identified biomarkers (LDA ≥ 4) is summarized in Supplementary Table S1. These biomarkers were primarily concentrated in the phyla Chloroflexi and Gemmatiomonadota, including genera such as g__*Subgroup_10*, g__unclassified_Chloroflexi, and g__unclassified_Gemmatiomonadota, indicating the most pronounced differences in bacterial community composition between the rhizosphere soil of *C*. *chinense* and other groups. The signature taxa in the CLG group were predominantly affiliated with Proteobacteria, such as g__*Novosphingobium* and g__*Paucibacter*, which were significantly enriched within *T*. *chinensis* root systems (Figure 6). The CLS group harbored significantly enriched taxa from Actinobacteriota and Proteobacteria, including orders o__Microtrichales and o__Xanthomonadales (Figure 6). Similarly, the ERTG group’s differential microbes were mainly Proteobacteria, with g__*Rhizobium* and g__*Sphingobium* significantly enriched (Figure 6), reflecting unique bacterial characteristics specific to *C*. *chinense* roots. In contrast, the JPS group had fewer differential taxa, with only moderately higher relative abundance of Bacteroidota, particularly order o__Flavobacteriales and family f__Flavobacteriaceae. The LWS group’s differential microbes were concentrated within the Gammaproteobacteria class of Proteobacteria, including salt-tolerant taxa g__*Truepera*, g__*Halomonas*, and order o__Ectothiorhodospirales that were significantly enriched (Figure 6). No significantly enriched biomarkers meeting the LDA ≥ 4 threshold were detected in the root samples of *S. salsa* (JPG) or *P. australis* (LWG).

### 3.6. Characteristics of the Bacterial Co-Occurrence Network

**Figure 7:**
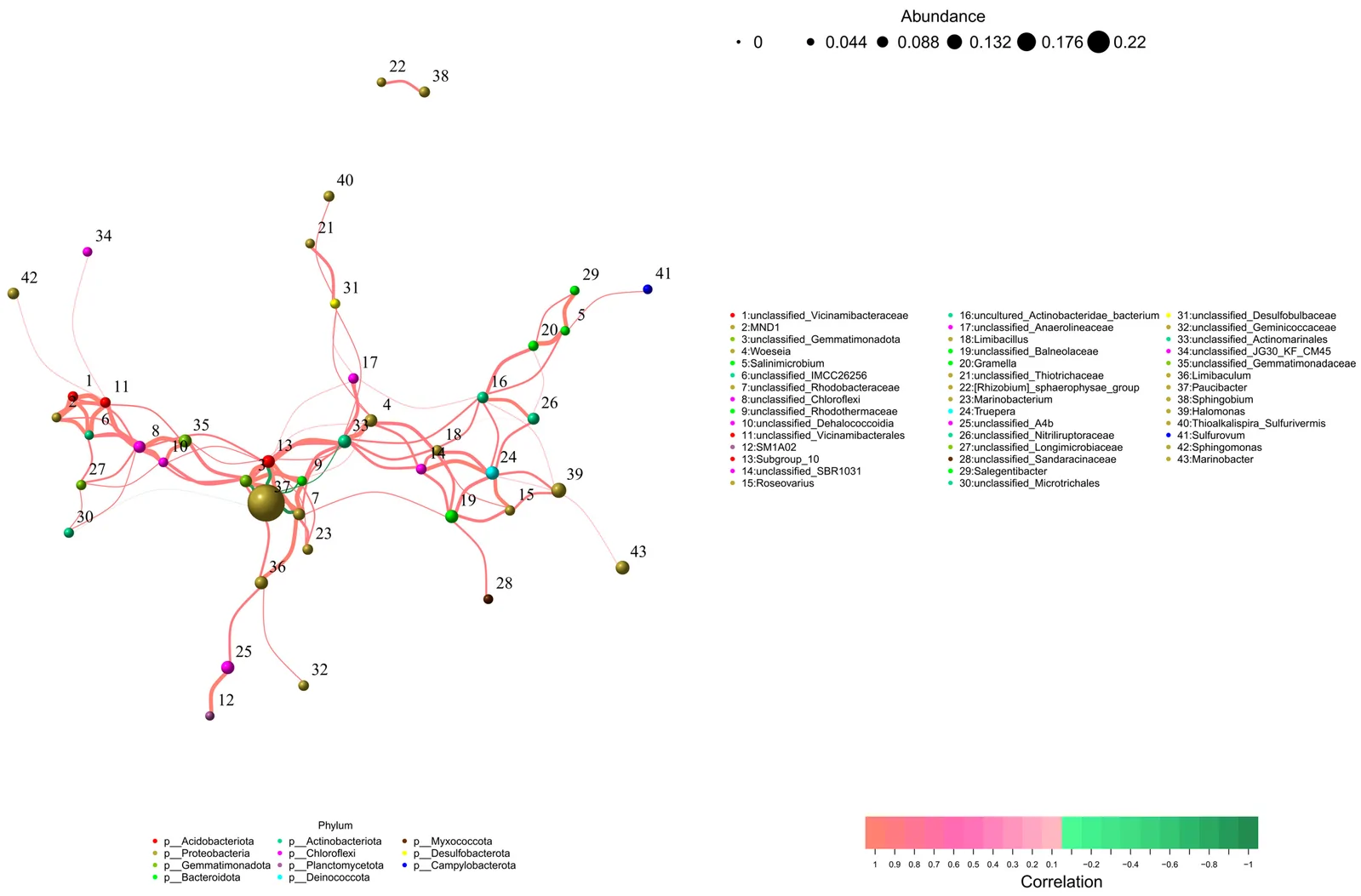
Co-occurrence network of bacterial genera derived from rhizosphere soil and root samples of the four halophyte species (*T. chinensis*, *P. australis*, *S. salsa*, and *C. chinense*). Each node represents a single bacterial genus, with node size proportional to its relative abundance across all samples, and node color indicating its affiliated phylum. Red edges stand for significant positive pairwise correlations, and green edges represent significant negative pairwise correlations. Full node degree data for all genera are summarized in Supplementary Table S2. Abbreviations: CLS = *T. chinensis* rhizosphere soil, CLG = *T. chinensis* root; LWS = *P. australis* rhizosphere soil, LWG = *P. australis* root; JPS = *S. salsa* rhizosphere soil, JPG = *S. salsa* root; ERTS = *C. chinense* rhizosphere soil, ERTG = *C. chinense* root.

Node 13 (*Subgroup_10*) functioned as one of the core keystone hubs in the co-occurrence network. It had a node degree of 11, which was among the highest values across all genera, indicating extensive direct correlations with other microbial taxa. Detailed node degree values for all detected genera are provided in Supplementary Table S2. The network exhibited a distinct modular structure (Figure 7): the left module was mainly composed of Acidobacteriota, Actinobacteriota, Chloroflexi and Gemmatimonadota, while the central module was dominated by Proteobacteria, Gemmatimonadota, Bacteroidota and Actinobacteriota, with a small proportion of Desulfobacterota and Campylobacterota. Bacterial taxa clustered clearly by phylum. Most pairwise correlations were positive (red edges), and negative correlations (green edges) were scarce. Strong positive correlations were widely detected within the same phyla, whereas cross-phylum negative correlations were rare. Comparative topological analysis revealed differences in average node degree and modularity between rhizosphere and root subnetworks. The average node degree and modularity of rhizosphere subnetworks were higher than those of root subnetworks. The ERTS subnetwork had the highest average node degree among all rhizosphere groups. *Subgroup_10* was identified as the core hub taxon in each rhizosphere subnetwork.

### 3.7. Bacterial Functional Potential Prediction Based on FAPROTAX

**Figure 8:**
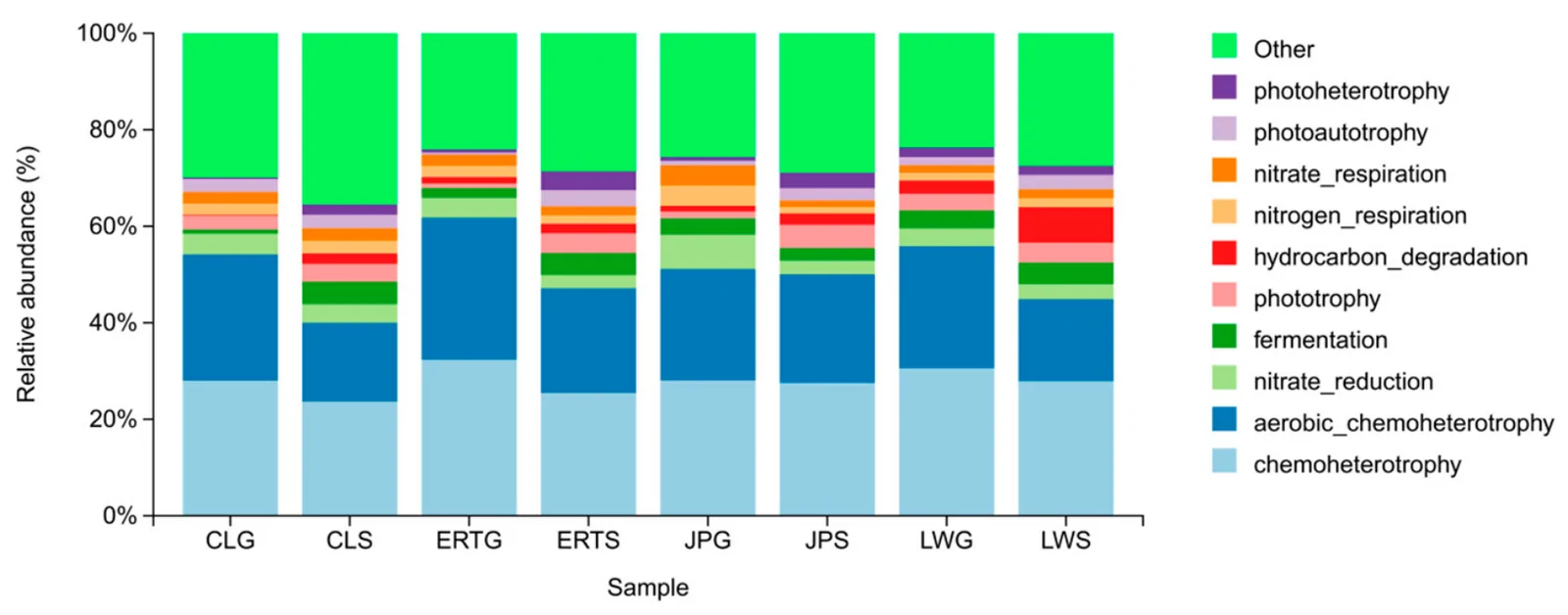
Stacked bar chart depicting the functional composition of bacterial communities across eight sample types: rhizosphere soils and roots of the four plant species (*T. chinensis*, *P. australis*, *S. salsa*, and *C. chinense*). The *y*-axis indicates relative abundance (as a percentage), and each colored segment corresponds to a distinct metabolic functional category. Clear compositional differences are observed between root and rhizosphere soil bacterial communities. Abbreviations: CLS = *T. chinensis* rhizosphere soil, CLG = *T. chinensis* root; LWS = *P. australis* rhizosphere soil, LWG = *P. australis* root; JPS = *S. salsa* rhizosphere soil, JPG = *S. salsa* root; ERTS = *C. chinense* rhizosphere soil, ERTG = *C. chinense* root.

Functional prediction based on 16S rRNA sequences annotated against the FAPROTAX database revealed obvious niche-specific divergence of bacterial metabolic potentials across eight sample compartments (Figure 8). The core functional traits across all samples were chemoheterotrophy and aerobic chemoheterotrophy, indicating that heterotrophic metabolism dominated energy acquisition by microbes at the root–soil interface in saline-alkaline environments. The “Other” miscellaneous functional group occupied variable proportions across groups, generally more abundant in rhizosphere soils than root tissues. In rhizosphere soils of the same plant species, phototrophic-related functions such as photoautotrophy, photoheterotrophy, and phototrophy, nitrogen metabolism functions including nitrate reduction, nitrate respiration, and nitrogen respiration, as well as fermentation and hydrocarbon degradation, showed relatively higher abundances than in corresponding root samples. Conversely, root samples exhibited greater abundances of chemoheterotrophy and aerobic chemoheterotrophy. Functional compositions varied among different plant species: the roots of *C. chinense* had the highest abundances of the two heterotrophic functions; reed and *S. salsa* rhizosphere soils showed higher abundances of nitrogen metabolism and hydrocarbon degradation functions; while *T. chinensis* roots and rhizosphere soils generally displayed lower abundances of rare metabolic functions. Visible compositional separation between root and rhizosphere compartments is clearly observed in the stacked bar chart.

**Figure 9:**
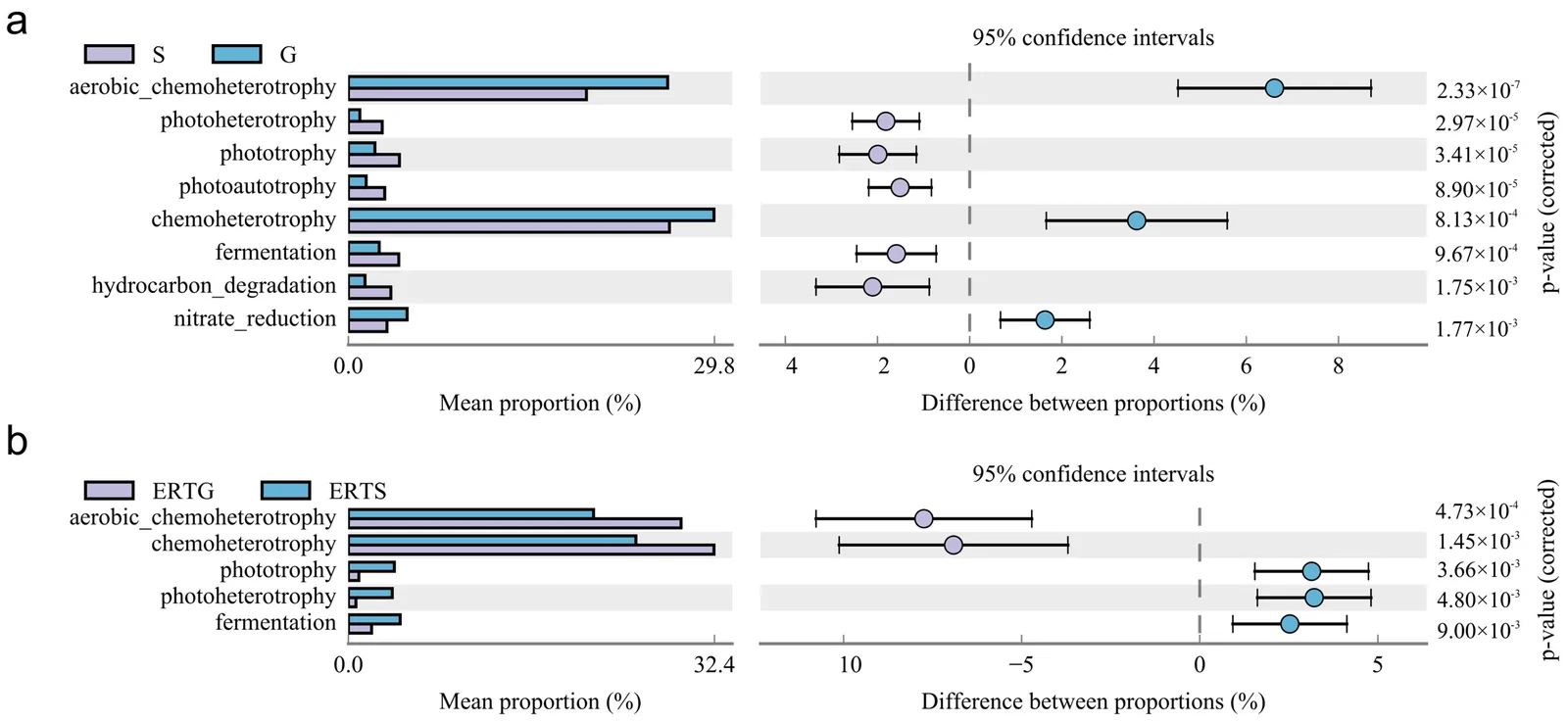
Pairwise comparison of bacterial functional pathways annotated by FAPROTAX. (**a**) Comparison between all root samples (G) and all rhizosphere-soil samples (S); (**b**) comparison between root tissue (ERTG) and rhizosphere soil (ERTS) of *C. chinense*. The bar charts on the left show the mean proportion of each functional pathway. Dots in the right panels represent the differences in proportions between groups, error bars indicate 95% confidence intervals, and values on the right are *p*-values adjusted by the false discovery rate (FDR) for multiple-test correction.

To statistically verify the visually apparent functional differentiation and meet the requirement for inter-group difference validation, pairwise comparative analyses were performed using the platform-supported test method at a significance threshold of 0.05, with all *p*-values adjusted by the false discovery rate (FDR) method for multiple testing (Figure 9). Two representative pairwise comparisons were selected to cover core research dimensions. Figure 9a presents the comparison between all pooled root samples and all pooled rhizosphere soil samples. Consistent with the stacked bar chart trend, aerobic chemoheterotrophy and chemoheterotrophy showed significantly higher relative abundance in root compartments, with confidence intervals entirely separated from zero and adjusted *p* < 0.001. By contrast, all phototrophic, nitrogen-reduction, fermentation and hydrocarbon-degradation pathways were significantly enriched in rhizosphere soils, demonstrating robust habitat-driven functional divergence at the global scale of all samples. Figure 9b displays the within-species pairwise contrast of *C. chinense* (ERTG vs. ERTS), the compartment pair with the most prominent taxonomic differentiation. Again, core heterotrophic functions were significantly enriched in root tissue, while phototrophy, photoheterotrophy and fermentation exhibited markedly higher proportions in corresponding rhizosphere soil. Collectively, the pairwise statistical comparisons quantitatively confirmed significant differences in predicted bacterial functional profiles between root endosphere and rhizosphere soil microhabitats, and consistent habitat-divergent predicted functional potential existed even within paired root–rhizosphere samples collected from the same halophyte individual.

## 4. Discussion

The overall differentiation of bacterial communities between root endosphere and rhizosphere soil observed in this study fully supports our first hypothesis that plant-associated microhabitats act as strong filters shaping bacterial assemblages; meanwhile, host-specific biomarker taxa and divergent functional profiles across four halophytes validate our second hypothesis on plant-species-specific rhizosphere bacterial assembly. This comparative investigation of rhizosphere–root bacterial communities across multiple co-occurring native halophytes provides valuable observational evidence for understanding commonalities and inter-species divergence of plant-bacterial adaptive associations and reflects microbial adaptive characteristics of local northern Yellow River Delta wetland plant communities [4].

This study reveals a consistent, taxonomically structured differentiation in bacterial community composition between the rhizosphere soil and root endosphere across four salt marsh plant species at both the phylum and genus levels. Proteobacteria dominated the root endosphere of all host plants, accounting for over 80% of the relative abundance in representative samples (e.g., *Tamarix chinensis* root tissue (CLG) and *Suaeda salsa* root tissue (JPG)). In contrast, Actinobacteriota, Bacteroidota, and Chloroflexi contained multiple taxa significantly enriched in rhizosphere compartments (Figure 6). At the genus level, *Paucibacter* showed consistent and statistically significant enrichment across all root endospheres, whereas unclassified members of Actinomarinales and Balneolaceae were preferentially associated with the rhizosphere. This spatial partitioning highlights the role of the root system as a strong selective biological filter: root exudates modulate the physicochemical properties of the rhizosphere microenvironment, while structural and immunological barriers within the root cortex and vascular tissues impose progressively stronger deterministic selection on bacterial colonization [6,19,20]. Notably, the rhizosphere of *Cynanchum chinense* (ERTS) harbored the highest amplicon sequence variant (ASV) richness (*n* = 9042) and alpha diversity among all sampled compartments, and its rarefaction curve approached the asymptote most steeply, indicating greater community saturation. These findings suggest that this pioneer halophytic herb may support a more complex and functionally diverse bacterial community through the secretion of abundant secondary metabolites, particularly steroidal saponins [14], thereby enhancing its ecological fitness during establishment in mildly saline-alkaline soils. Overall, rhizosphere and endophytic bacterial communities differed significantly in composition and structure, with host plant life form acting as a key determinant [2,21].

Accordingly, while we interpret FAPROTAX-derived profiles as taxon-inferred functional potential rather than direct metabolic measurements, the observed host-specific enrichments—particularly the coupling of nitrogen metabolism with aerenchyma-bearing *S. salsa* and *P. australis*—are strongly consistent with their well-documented physiological traits [2,21]. This concordance supports the validity of using 16S-based functional predictions as an effective preliminary approach for generating ecological hypotheses in coastal wetland studies [8,9,22,23]. The reduced functional diversity observed in the *T. chinensis* rhizosphere, corresponding with its salt-excretion strategy that creates a low-organic-carbon niche, further reinforces the view that halophytes actively sculpt the “functional second genome” of their associated microbiomes through distinct life-form-specific root traits.

Moreover, the 16S rRNA gene-based approach does not distinguish active from dormant populations, nor capture growth rates or r/K-strategist traits that influence community assembly and recovery dynamics [24]. Our single-time-point survey primarily reflects a mid-term assembly state rather than full successional trajectories. Nonetheless, this dataset provides a foundational taxonomic and co-occurrence framework against which future time-series sampling can assess shifts in community composition and functional resilience during long-term wetland restoration [8,11,22,24].

LEfSe analysis identified several plant-specific bacterial biomarkers. *Paucibacter* and *Novosphingobium* were significantly enriched in the root endosphere of *T. chinensis* (Figure 6); both taxa are known to possess ACC deaminase activity, a key mechanism for alleviating salt-induced physiological stress. *Subgroup_10* (affiliated with Chloroflexi) and unclassified members of Gemmatimonadota were significantly enriched in the rhizosphere of *C. chinense* (Figure 6), whereas *Halomonas* and *Truepera*, two well-documented halotolerant genera, were uniquely abundant in the *P. australis* rhizosphere. Co-occurrence network analysis further revealed that *Subgroup_10* acted as a topological hub, exhibiting strong positive correlations with Acidobacteriota, Actinobacteriota, and other phylogenetically diverse lineages. This central position implies a potential keystone role for *Subgroup_10* in maintaining bacterial community resilience and functional stability under saline-alkaline stress. Further grouped subnetwork comparison revealed consistent habitat-driven differentiation in microbial interaction patterns: rhizosphere-associated subnetworks showed higher connectivity and modularity relative to root endosphere subnetworks, indicating more intricate interspecies associations in rhizosphere microhabitats. Meanwhile, *Subgroup_10* universally served as the core hub across all four plant rhizosphere subnetworks, whereas no shared keystone genus existed in root compartments, highlighting that host filtering effects diverge sharply between rhizosphere and endosphere niches during community assembly. Collectively, our results demonstrate taxonomic differences and distinct interaction patterns among bacterial assemblages associated with different halophyte–microhabitat combinations. Plant–microbe–soil feedback effects during succession remain to be validated by further manipulative experiments. Our dataset could offer preliminary observational references for exploring native-halophyte-associated bacterial assembly in coastal saline-alkaline wetlands [8,11].

All sampling plots were situated in the northern Yellow River Delta Binzhou coastal salt-marsh wetland. Undisturbed reference sites with fully matched edaphic and hydrological backgrounds are difficult to obtain in this coastal zone [23]. Given this practical constraint, our comparison among four co-existing pioneer halophytes provides a critical baseline for understanding host-driven microbial assembly in coastal saline-alkaline wetlands [8]. This study provides a high-resolution taxonomic and co-occurrence framework for root-associated bacterial communities of native halophytes [8,11]. Our identification of habitat-specific keystone taxa (e.g., *Subgroup_10*) and host-dependent interaction networks offers testable hypotheses for future manipulative experiments [25]. In subsequent long-term monitoring, we will integrate multi-season paired sampling with newly established reference plots to systematically track dynamics of root-associated microbiomes at the local wetland [26]. Our sampling plots are located in Binzhou coastal salt-marsh, an area historically subjected to *S. alterniflora* removal. Our findings provide insights for future studies concerning microbiome-associated investigations in this coastal wetland.

## 5. Conclusions

This study investigates host-driven bacterial community assembly of four representative native halophytes (*P. australis*, *S. salsa*, *T. chinensis*, *C. chinense*) in the Binzhou coastal salt-marsh wetland of the northern Yellow River Delta. We systematically compare the structural composition, taxonomic diversity, and functional profiles of bacterial communities inhabiting two distinct plant-associated compartments: rhizosphere soil and the root endosphere. Our multi-species comparisons reveal both common features and host-specific divergence of plant-associated bacterial assemblages, which reflect plant-microbe adaptive characteristics of local saline-alkaline wetland ecosystems. Our results show that rhizosphere bacterial communities consistently exhibit higher α-diversity (encompassing species richness, evenness, and Shannon diversity), greater compositional reproducibility across replicates, and broader metabolic capacities, including nitrogen cycling, photosynthetic pigment biosynthesis, and hydrocarbon catabolism, compared with endosphere communities. In contrast, the endosphere harbors highly specialized, host-filtered assemblages dominated by chemoheterotrophic bacteria, with pronounced phylogenetic and functional divergence among plant hosts. Moreover, plant species differentially shape rhizosphere bacterial community assembly trajectories and microbial co-occurrence patterns. Topological comparison of subnetworks demonstrated that rhizosphere bacterial networks possessed higher connectivity and modularity than root networks, with the *C. chinense* rhizosphere harboring the most complex interspecies interactions and the universal keystone taxon *Subgroup_10*. *C. chinense* supports the highest bacterial α- and β-diversity and is uniquely enriched in Chloroflexi and Gemmatimonadota, whereas *T. chinensis* selectively enriches *Paucibacter* and other taxa with documented plant growth-promoting traits. (e.g., phosphate solubilization, auxin synthesis). These patterns highlight that plant life-history strategies and key functional traits, such as salt-excreting glandular structures, deep taproot architecture, and tolerance to saline-alkaline stress, act as primary determinants of bacterial community assembly. Collectively, this work elucidates the critical role of native pioneer halophytes in reconfiguring biogeochemical processes at the rhizosphere–soil interface. It thereby provides tentative plant-microbe-associated observational insights to support precision restoration research for the northern Yellow River Delta saline-alkaline wetland.

## Data Availability

Raw 16S rRNA amplicon sequencing reads are available in the NCBI Sequence Read Archive (SRA) under BioProject accession PRJNA1513472, covering 80 BioSample entries (SAMN62451007-SAMN62451086) and corresponding SRA entries (62451007-62451086). Other datasets generated and analysed during this study are available in the Zenodo repository: https://doi.org/10.5281/zenodo.21770044.

## Supplementary Materials

The following supporting information can be downloaded at: https://www.mdpi.com/article/10.3390/microorganisms14092021/s1, Table S1: List of bacterial biomarkers identified by LEfSe analysis (LDA ≥ 4); Table S2: Node-level characteristics of genera in the bacterial co-occurrence network.

## References

[1] Zhi L., Li X., Li X., Ma T., Guo W., Cui B., Wang D. (2026). An integrated strategy maximises cobenefits of conservation and restoration for ecosystem services in coastal wetlands. Commun. Earth Environ..

[2] Pang B., Xie T., Ning Z., Cui B., Zhang H., Wang X., Gao F., Zhang S., Lu Y. (2023). Invasion patterns of *Spartina alterniflora*: Response of clones and seedlings to flooding and salinity-A case study in the Yellow River Delta, China. Sci. Total. Environ..

[3] Shang S., Li L., Zhang Z., Zang Y., Chen J., Wang J., Wu T., Xia J., Tang X. (2022). The Effects of Secondary Growth of *Spartina alterniflora* after Treatment on Sediment Microorganisms in the Yellow River Delta. Microorganisms.

[4] Sun P., Wu Y., Zhu P., Wang J., Yu X., Guo W. (2025). *Spartina alterniflora* invasion significantly alters the assembly and structure of soil bacterial communities in the Yellow River Delta. Front. Microbiol..

[5] Li H., Cui G., Liu H., Wang Q., Zhao S., Huang X., Zhang R., Jia M., Mao D., Yu H. (2025). Dynamic Analysis of *Spartina alterniflora* in Yellow River Delta Based on U-Net Model and Zhuhai-1 Satellite. Remote Sens..

[6] Afridi M.S., Fakhar A., Kumar A., Ali S., Medeiros F.H., Muneer M.A., Ali H., Saleem M. (2022). Harnessing microbial multitrophic interactions for rhizosphere microbiome engineering. Microbiol. Res..

[7] Chepsergon J., Moleleki L.N. (2023). Rhizosphere bacterial interactions and impact on plant health. Curr. Opin. Microbiol..

[8] Solomon W., Janda T., Molnár Z. (2024). Unveiling the significance of rhizosphere: Implications for plant growth, stress response, and sustainable agriculture. Plant Physiol. Biochem..

[9] Jefferson T.A., Alizadeh M., Prismantoro D., Albert M., Wulandari I., Miranti M., Doni F. (2026). The microbiome as the second genome: Engineering the phytobiome for climate-resilient agriculture. J. Plant Interact..

[10] Mishra S., Zhang X., Yang X. (2024). Plant communication with rhizosphere microbes can be revealed by understanding microbial functional gene composition. Microbiol. Res..

[11] Tang Z., Tan W., Li R., Weng L., Chen X., Xi B., Lv D. (2025). Advances in Rhizosphere Microbiome and Rhizosphere Immunity Effect: A Review. J. Agric. Food Chem..

[12] Hussain M., Adeel M., White J.C. (2025). Nano-selenium: A novel candidate for plant microbiome engineering. Trends Plant Sci..

[13] Ding M., Osayande I.S., Tsuda K. (2024). Selenium nanoboosting of plant-beneficial microbiome. Cell Host Microbe.

[14] Li P., Liu J., Saleem M., Li G., Luan L., Wu M., Li Z. (2022). Reduced chemodiversity suppresses rhizosphere microbiome functioning in the mono-cropped agroecosystems. Microbiome.

[15] Chroston E.C.M., Bziuk N., Stauber E.J., Ravindran B.M., Hielscher A., Smalla K., Wittstock U. (2024). Plant glucosinolate biosynthesis and breakdown pathways shape the rhizosphere bacterial/archaeal community. Plant Cell Environ..

[16] Fan X., Ge A.-H., Qi S., Guan Y., Wang R., Yu N., Wang E. (2025). Root exudates and microbial metabolites: Signals and nutrients in plant-microbe interactions. Sci. China Life Sci..

[17] Xun W., Liu Y., Ma A., Yan H., Miao Y., Shao J., Zhang N., Xu Z., Shen Q., Zhang R. (2024). Dissection of rhizosphere microbiome and exploiting strategies for sustainable agriculture. New Phytol..

[18] Dwivedi S.L., Vetukuri R.R., Kelbessa B.G., Gepts P., Heslop-Harrison P., Araujo A.S.F., Sharma S., Ortiz R. (2025). Exploitation of rhizosphere microbiome biodiversity in plant breeding. Trends Plant Sci..

[19] Huang Y., Huang Y., Hou J., Wu L., Christie P., Liu W. (2023). Microbial community assembly of the hyperaccumulator plant *Sedum plumbizincicola* in two contrasting soil types with three levels of cadmium contamination. Sci. Total. Environ..

[20] Hua M., Jiang H., Yuan R., Kong J. (2026). Diversity and composition of root-associated fungal communities in critically small population of *Cypripedium subtropicum*. Front. Plant Sci..

[21] Shang S., Hu S., Liu X., Zang Y., Chen J., Gao N., Li L., Wang J., Liu L., Xu J. (2022). Effects of *Spartina alterniflora* invasion on the community structure and diversity of wetland soil bacteria in the Yellow River Delta. Ecol. Evol..

[22] Hu J.-P., He Y.-Y., Li J.-H., Lü Z.-L., Zhang Y.-W., Li Y.-H., Li J.-L., Zhang M.-X., Cao Y.-H., Zhang J.-L. (2024). Planting halophytes increases the rhizosphere ecosystem multifunctionality via reducing soil salinity. Environ. Res..

[23] Li L., Cheng K., Du Y., Zhang Y., Zhou Y., Jin Y., He X. (2025). Rhizosphere Microbes from *Populus euphratica* Conferred Salt Stress Resistance to *Populus alba* × *Populus glandulosa*. Plant Cell Environ..

[24] Yang J., Cui L., Zhuo Z., Tian J., Li P., Min Y., Ke Y. (2025). Rapid change in structural stability of tidal flats in response to large-scale eradication of *Spartina alterniflora*: Evidence from dense time-series PlanetScope satellite imagery. J. Environ. Manag..

[25] Gallego-Tévar B., Grewell B.J., Figueroa E., Castillo J.M. (2020). The role of exotic and native hybrids during ecological succession in salt marshes. J. Exp. Mar. Biol. Ecol..

[26] Iram N., Ren Y., Zhao R., Zhao S., Dong C., Han Y., Zhang Y. (2025). Deciphering Soil Keystone Microbial Taxa: Structural Diversity and Co-Occurrence Patterns from Peri-Urban to Urban Landscapes. Microorganisms.

